# Ultrafast exciton quenching by energy and electron transfer in colloidal CdSe nanosheet–Pt heterostructures†
†Electronic supplementary information (ESI) available: Synthesis details, transient absorption set-ups, NS and NS–Pt spectra fitting, kinetics fitting model and parameters, details about the exciton diffusion controlled energy transfer model. See DOI: 10.1039/c4sc02994a



**DOI:** 10.1039/c4sc02994a

**Published:** 2014-11-04

**Authors:** Kaifeng Wu, Qiuyang Li, Yongling Du, Zheyuan Chen, Tianquan Lian

**Affiliations:** a Department of Chemistry , Emory University , Atlanta , Georgia 30322 , USA . Email: tlian@emory.edu

## Abstract

Two-dimensional (2-D) semiconductor nanomaterials are receiving tremendous research interests due in part to their attractive light absorption and charge transport properties. Integration of catalytic metal nanoparticles with these 2-D semiconductors can potentially lead to new photocatalytic nanoheterostructures for efficient solar-to-fuel conversion. Here we report the synthesis and transient absorption study of colloidal quantum confined CdSe nanosheets with a Pt nanoparticle at the edge or vertex. Due to the large in-plane exciton mobility, ∼86.6 ± 0.5% of excitons generated in CdSe sheets can be transported to NS–Pt interface and quenched by energy transfer to Pt (with a half-life <150 fs). The remaining excitons (13.4 ± 0.5%) become localized due to fast hole trapping and can be dissociated by interfacial electron transfer to Pt (with a half life of ∼9.4 ± 0.7 ps). The resulting charge-separated states (with electrons in Pt and trapped holes in CdSe) are long-lived (half life of ∼75 ± 14 ns), suggesting possible applications for solar driven H_2_ generation.

## Abstract 2

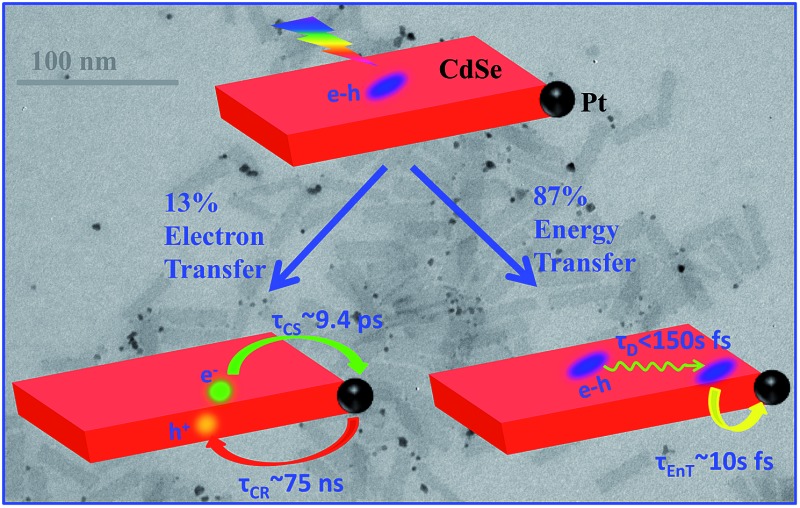
Large in-plane exciton mobility in CdSe nanosheets leads to ultrafast exciton quenching by energy transfer to Pt.

## Introduction

**Fig. 1:**
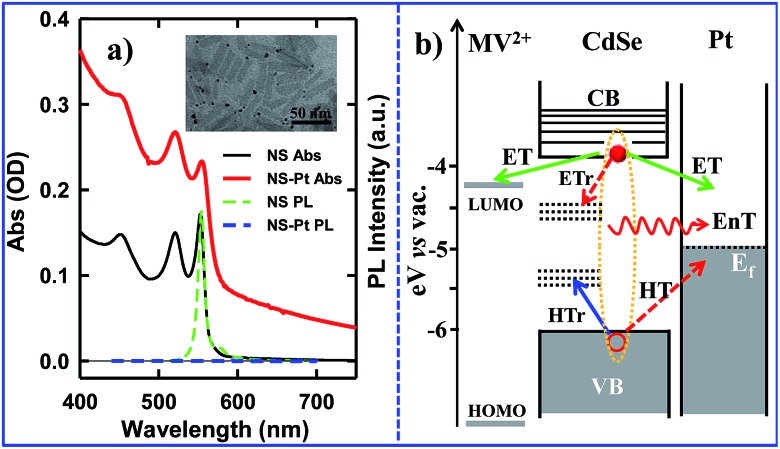
a) Absorption and photoluminescence (PL) spectra of CdSe NS (black solid line, green dashed line) and CdSe NS–Pt heterostructures (red solid line and blue dashed line). Inset: a representative TEM image of NS–Pt. (b) Schematic energy levels and possible exciton quenching pathways in NS–Pt and NS–MV^2+^ complexes. In addition to radiative e–h recombination, electron trapping (ETr) and hole trapping (HTr) pathways within the CdSe NS, excitons can be quenched through interfacial electron transfer (ET), hole transfer (HT) and energy transfer (EnT) to Pt. In contrast, ET is the only quenching pathway in NS–MV^2+^ complexes.

Colloidal quantum confined semiconductor–metal nanoheterostructures that combine tunable light absorption of the semiconductor domain and catalytic activity of metal nanoparticles are promising materials for solar energy conversion.^1–4^ To date, heterostructures of 0-dimenisonal (quantum dot or QD) and 1-dimensional (nanorods or NR, nanowires) semiconductors have been prepared and studied.^5–20^ More recently, 2-dimensional (2-D) semiconductor nanomaterials are receiving tremendous interests due to their attractive light absorption and charge transport properties.^21,22^ Among them are colloidal CdSe nanosheets (NSs) with atomically precise thicknesses of only a few CdSe layers.^23–28^ In addition to strong quantum confinement in the thickness direction, these nanosheets have large absorption cross sections,^29^ high photoluminescence (PL) quantum yields,^25,30^ and fast in-plane carrier transport,^30–32^ making them promising light-absorbing materials for photocatalysis. However, in semiconductor–metal heterostructures such as CdSe NS–Pt (Fig. 1b), the band alignment between CdSe NSs and Pt nanoparticles allows exciton quenching through electron, hole and energy transfers to the Pt domain, whereas only the electron transfer channel is desirable for solar-driven H_2_ generation. Our previous study of CdS–Pt NRs has shown that ultrafast exciton trapping suppresses the energy transfer pathway and favors exciton dissociation by electron transfer to Pt, making them interesting materials for solar-driven H_2_ generation.^20^ Unlike NRs, the atomically precise thickness of NSs may lead to ultrafast in-plane exciton motion and alter the exciton quenching mechanisms. For this reason, these NS–Pt heterostructures serve as an interesting model system for fundamental understanding of the interaction between excitons in 2-D nanomaterials with metal nanoparticles.

Herein, we report the synthesis and ultrafast spectroscopic study of Pt tipped colloidal CdSe NSs. We found that excitons in NSs were completely (∼100%) quenched by the Pt tip. However, 86.6% of excitons were quenched on an ultrafast time scale (half-life <150 fs) by energy transfer to Pt due to both fast in-plane exciton transport and fast energy transfer to Pt. The remaining ∼13.4% of excitons were trapped and then dissociated (with a half life of ∼9.4 ps) by electron transfer (ET) to Pt to form long-lived charge separated states (with a half life of ∼75 ns). Our results indicate that low charge separation yield is an efficiency limiting factor for the applications of this heterostructure to photocatalysis and point to potential approaches for improving their performances.

## Sample preparation and characterization

CdSe NSs were synthesized according to literature procedures.^23–25^ Pt deposition on nanosheets was achieved by thermal reduction of Pt(ii) acetylacetonate in the presence of oleic acid and oleylamine as both reducing reagents and capping ligands, similar to the reported procedure of Pt deposition on CdS nanorods.^7^ Details of sample syntheses can be found in the ESI.† Large area transmission electron microscopy (TEM) images of CdSe NSs before and after Pt tip growth are shown in Fig. S1.† These NSs typically exhibited rectangular morphology with average dimensions of 39.2 (±4.9) nm × 11.3 (±2.0) nm. Their thickness was estimated to be ∼1.52 nm from their absorption spectrum (see below). The average dimensions of NSs with Pt nanoparticle (3.1 ± 1.2 nm in diameter) were 39.9 (±5.8) nm × 10.9 (±1.6) nm, showing negligible etching of NSs during the Pt deposition process. Interestingly, instead of the highly-exposed (001) basal plane,^24^ the procedure selectively deposited only one Pt tip at either the edge or vertex of the rectangular NS (Fig. 1a inset), which likely resulted from high surface energies or sparse ligand coverage at these sites.^30^ Similar preferential deposition of Au nanoparticles at the edge over the basal plane of the CdSe nanobelts has been observed in a previous study.^30^ However, in that system, many Au nanoparticles were deposited on the edges of each nanobelt. The reason for this difference is not clear, but likely due to different metal precursor concentrations used in the synthesis. There existed a broad size distribution of the Pt nanoparticles deposited on our NSs, which, in principle, can lead to a large heterogeneity in quenching kinetics of NSs by Pt nanoparticles, as will be discussed later.

The absorption and photoluminescence (PL) spectra of CdSe NS and CdSe NS–Pt are compared in Fig. 1a. The CdSe NS exhibited sharp and discrete transitions arising from quantum confinement in the thickness direction.^23–25^ The two lowest energy peaks at 553 nm and 521 nm can be attributed to transitions to *n* = 1 conduction band (CB) electron level from *n* = 1 valence band (VB) heavy hole (hh → e) and *n* = 1 light hole (lh → e) levels, respectively.^23^ The width of (hh → e) absorption band was only ∼35 meV (Fig. S2, Table S1†), indicating a uniform thickness. These transition energies corresponded to a thickness of ∼1.52 nm (2.5 times Zinc Blende CdSe lattice constant or 5 monolayers of CdSe).^33^ The absorption spectrum of CdSe NS–Pt showed a broad feature extending from UV to the near IR in addition to the CdSe exciton bands. Detailed analysis (Fig. S2, Table S1†) showed that the broad feature was the same as free Pt particles and could be attributed to the strong d–sp interband transition of Pt nanoparticles.^34^ In addition, the sharp CdSe exciton peaks were slightly red-shifted and broadened compared to free CdSe Ns. PL of CdSe NSs was dominated by a sharp band edge emission centered at 554 nm with a small Stokes shift of ∼4 meV and a quantum yield (QY) of 36%, consistent with previous reports.^25^ In CdSe NS–Pt, the PL of NS was completely quenched, indicative of strong electronic interaction between the CdSe NS and Pt particle. It also indicates that all the NSs are in contact with Pt particles, although some of these particles are too small to be clearly observed in the TEM image shown in Fig. S1.† In addition, there exists a possibility of PL quenching by surfaced adsorbed Pt(0) or Pt(ii) species, similar to previously observed CdSe/CdS nanorods quenching by Au precursors used for Au nanoparticle deposition.^35^

## Ultrafast spectroscopic studies

To examine the potential of this NS–Pt heterostructure as a photocatalyst, we investigated the quenching mechanisms using pump–probe transient absorption (TA) spectroscopy. As a comparison and to facilitate spectroscopic assignment, we also studied the exciton quenching in NS–methylviologen (MV^2+^) complexes, in which only the ET pathway is energetically allowed (Fig. 1B).

**Fig. 2:**
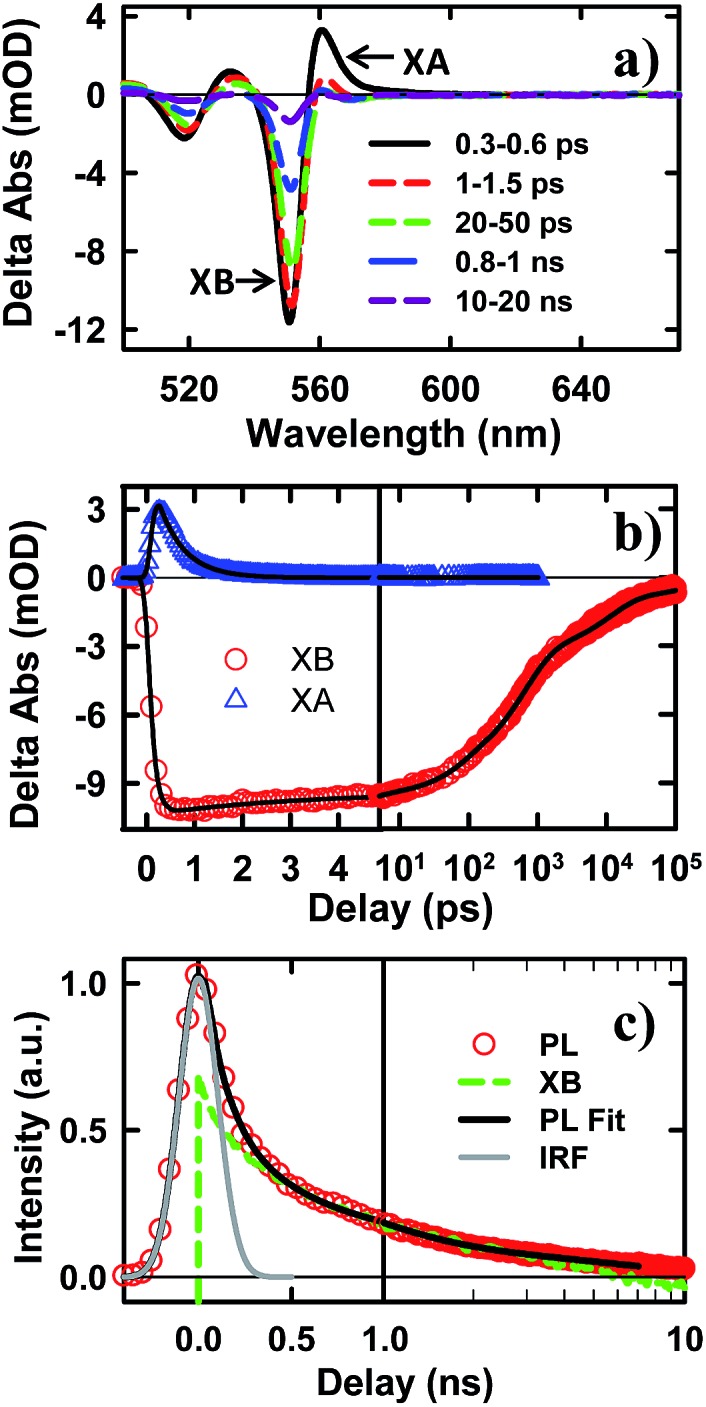
TA spectra and kinetics of CdSe NSs measured with 400 nm excitation. (a) TA spectra of NSs at indicated time. (b) Kinetics of XA (blue triangles) and XB (red circles) in NSs and their fits (black solid lines). (c) Comparison of XB bleach (green dashed line) and PL decay of NSs (red circles). XB is scaled and vertically shifted for comparison. Also shown are the IRF of PL decay experiment (gray line) and a fit to the PL decay (black solid line).

**Fig. 3:**
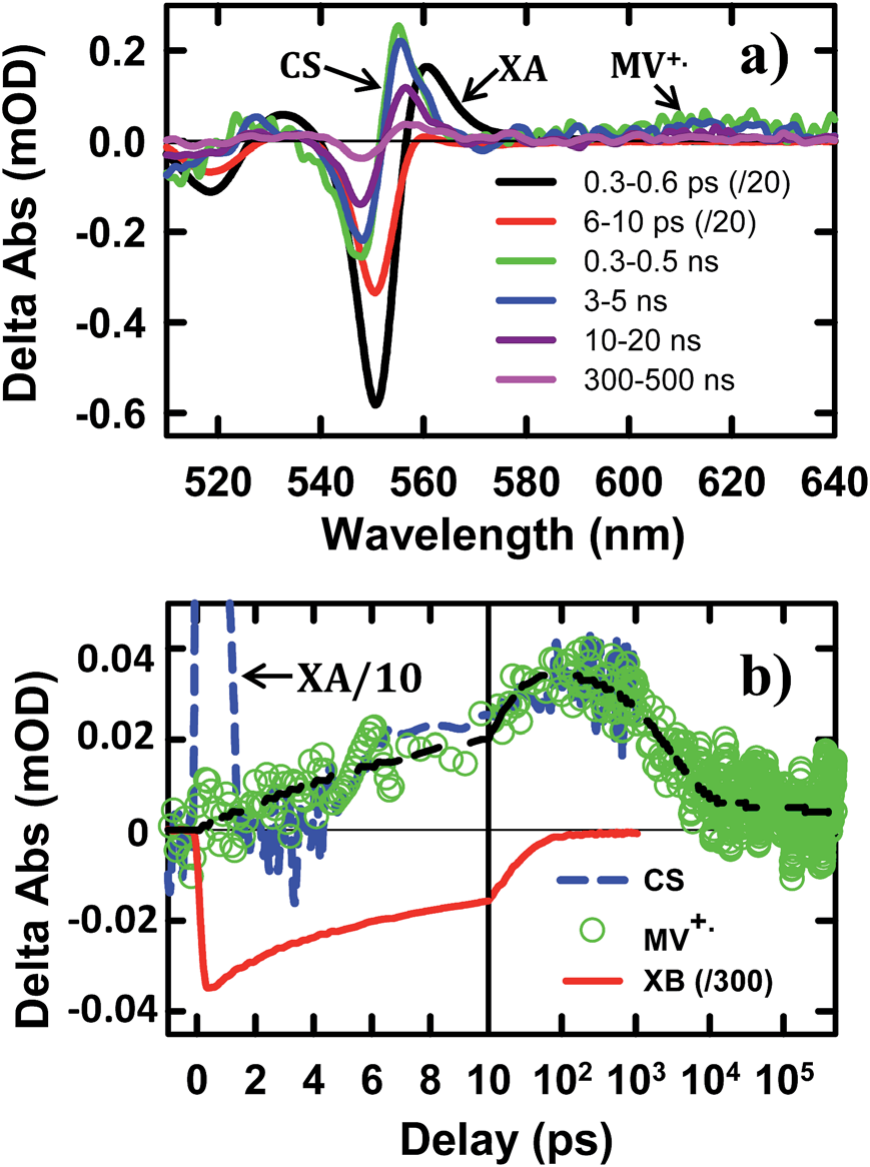
TA spectra and kinetics of CdSe NS–MV^2+^ complexes measured with 400 nm excitation. (a) TA spectra evolution from 0.3 ps to 500 ns. The first two spectra have been reduced by a factor of 20 for better comparison. (b) Kinetics of MV^+^˙ radical (green circles) and the complementary NS XB (red solid line) and XA/CS signal (blue dashed line). The latter have been scaled for better comparison. The black dashed line is a multi-exponential fit to the formation and decay kinetics of MV^+^˙ radical signal.

We first examine carrier dynamics and their transient spectral signatures in free CdSe NSs. The transient absorption (TA) spectra of CdSe NSs (Fig. 2a) measured with 400 nm excitation show an exciton bleach (XB) feature at ∼552 nm and an exciton absorption (XA) feature at ∼560 nm. In this measurement we have used low excitation flux to ensure that the signal is dominated by NSs with single excitons (see Fig. S3, ESI† for details), excluding the complications of multi-exciton annihilation dynamics. Detailed assignments of these TA features in NSs have yet to be reported, although XA and XB features have been observed in a previous study of multiple exciton dynamics of NSs,^36^ and are well understood in QDs and NRs.^37–41^ As will be discussed below (Fig. 3), the XB feature in CdSe NSs is dominated by CB electron state filling induced bleach of the lowest energy exciton band and can be used to follow the dynamics of the CB electron. The XB feature in free NSs (Fig. 2c) grew in with a time constant of 80 ± 27 fs, corresponding to the time scale of hot electron relaxation from the excited level to the CB *n* = 1 level. Fitting the XB decay by multiple exponential functions requires four components (see Table S3†).

In addition to state-filling XB signals, the presence of an exciton also shifts the energy of all exciton transitions due to exciton–exciton interaction, giving rise to the XA feature in the TA spectra.^37^ Because of the state-specific exciton–exciton interaction strength, the XA feature provides a sensitive probe of the initial exciton relaxation dynamics.^41,42^ The XA feature in free CdSe NS forms instantaneously (≪150 fs, IRF) and decays with a time constant of 0.41 ± 0.12 ps (Fig. 2c). In QDs and NRs, this decay leads to the growth of XB formation, and can be attributed to the cooling of electrons from the higher levels to the band edge.^37,43,44^ In contrast, in CdSe NSs, the decay of XA signal is much slower than the growth of the XB signal (80 ± 27 fs). Furthermore, the TA spectra at early-time (Fig. S5†) showed a redshift of XA and XB features during XA decay, indicting a decrease of bi-exciton interaction but not the population of electrons at the lowest energy (*n* = 1) CB level. We tentatively attribute this XA decay to in-plane exciton localization (*i.e.* the loss of extra kinetic energy of in-plane motion) and trapping processes (see ESI S8† for details).^37,43,44^

While the XB recovery probes the electron decay dynamics from the *n* = 1 CB level, band edge PL decay depends on both the CB electron and VB hole. The XB recovery and band-edge PL decay agree well on the ∼300 ps to 3 ns time scale (Fig. 2d), consistent with previously-reported lifetime of band edge excitons in related NSs.^25^ The slower electron (XB) decay components (10.9 ± 0.3 ns and 220 ± 8 ns with 32.4 ± 0.5% of the total XB amplitude, Table S3†) are much longer than the band edge PL decay, suggesting an electron decay process that does not lead to band edge PL. This can be attributed to electron recombination with trapped holes, which is typically slower due to reduced electron–hole overlap.^20^ The PL decay shows an instrument response (∼240 ps) limited fast decay component with an amplitude that is much larger than XB recovery on the same time scale, suggesting the presence of ultrafast hole decay (Table S3†). The comparison of the TA and PL results suggests that in the NS ensemble, 32.4 ± 0.5% of excitons undergo fast hole trapping, which leads to the long-lived (>3 ns) XB (CB electron) signal and the fast decay (<240 ps) in the PL signal. The remaining 67.6 ± 0.5% excitons decay *via* fast radiative and nonradiative recombination on the <3 ns time scale.

To help assign the XB spectral signature and to identify the TA spectral signature of charge separated states, we have also examined the charge separation and recombination processes in CdSe NS–MV^2+^ complexes, in which photo-reduction of MV^2+^ forms MV^+^˙ radicals with distinct absorption band at ∼610 nm. The static absorption spectrum of CdSe NS–MV^2+^ complexes (Fig. S4†) shows the absorption of MV^2+^ at ∼260 nm in addition to NS bands. In the TA spectrum of CdSe NS–MV^2+^ complexes (Fig. 3a), the XB feature, generated upon 400 nm excitation of the NS, completely recovered in 200 ps and the resultant spectrum contained derivative-like signals of NS exciton bands and a positive absorption band of MV^+^˙ radicals at ∼610 nm. The decay kinetics of XB (Fig. 3b) agree well with the formation kinetics of MV^+^˙ radicals, suggesting that excitons in CdSe NSs decay by electron transfer to MV^2+^. It also confirms that the XB feature is due solely to the state filling of the CB electron level with negligible contribution of the VB holes, consistent with previous observations in 0D (QD)^37,45,46^ and 1D (NR)^20,43,44^ II–VI nanocrystals. The derivative-like feature of NS exciton bands can be attributed to the effect of charge separation on the exciton, caused by either the hole in the CdSe NS and/or the electric field of the separated charges (NS^+^–MV^+^˙). As shown in Fig. 3b, the CS formation kinetics follows that of MV^+^˙ radical formation and XB bleach recovery. The subsequent decay of the CS and MV^+^˙ radical signals can be attributed to the charge recombination process. Therefore, the CS signal provides a convenient probe for the formation and decay of the charge separated state, especially in systems where the reduced electron acceptors lack clear spectral signatures (such as NS–Pt complexes to be described below). It should be noted that the CS signal resembles the XA feature because both result from the shift of exciton bands caused by interaction with additional charges/excitons. However, as shown in Fig. 3, these features have different red-shift amplitudes (due to different interaction strengths) and different formation and decay kinetics (the XA signal decays within the first ps).

**Fig. 4:**
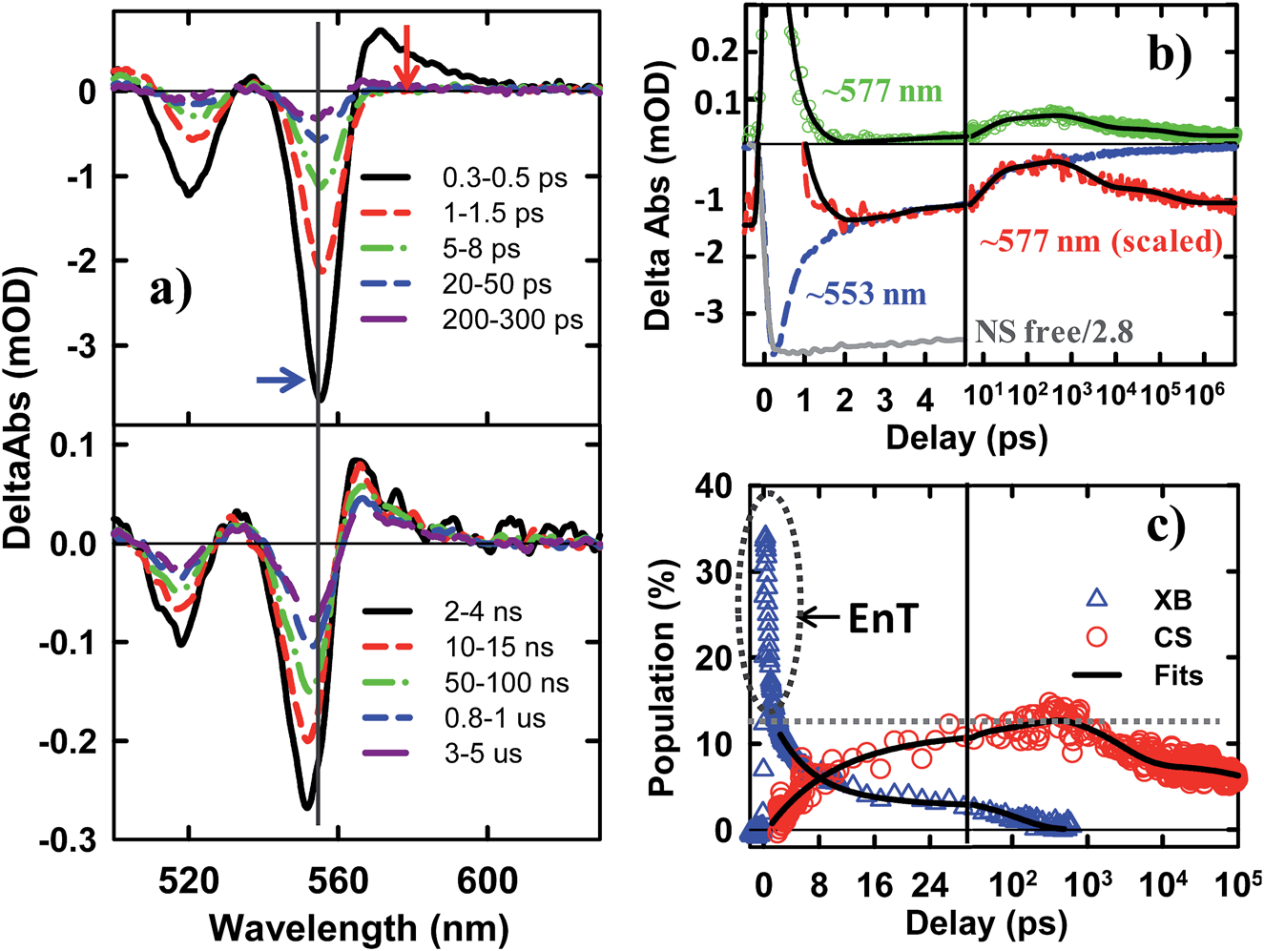
TA spectra and kinetics of CdSe NS–Pt measured with 400 nm excitation. (a) TA spectra at indicated time delays from 0.3 ps to 300 ps (upper panel) and from 2 ns to 5 us (lower panel). (b) Comparison of TA kinetics at ∼553 nm (XB, averaged between 551–555 nm, blue dashed line) and ∼577 nm (average between 573 and 580 nm, green circles). Also shown is the kinetics at 577 nm that has been scaled and displaced vertically (red dashed line). Kinetics at ∼553 nm in free NS (gray solid line) has been reduced by a factor of 2.8 for better comparison. The black solid line is a fit to kinetics at ∼577 nm. (c) Time-dependent population of XB (blue triangles) and charge separated states (CS, red circles). The black solid lines are multi-exponential fits to them. The gray dashed line indicates the efficiency of exciton dissociation (13.4 ± 0.5%). The gray dashed circle indicates ultrafast exciton quenching by energy transfer pathways. In (b) and (c), the delay time axes are in linear scale at early delay times and in logarithmic scale at longer decay times.

The TA spectra of NS–Pt after 400 nm excitation are shown in Fig. 4a. Our previous study on NR–Pt heterostructures shows that Pt nanoparticles can absorb 400 nm light and reduce the number of photons absorbed by the NSs, although the excited Pt do not show any TA spectral signatures in the visible region.^20^ To account for this effect, we scaled the TA signal of NS–Pt to correspond to the same number of absorbed photons by the NS in both samples (see ESI S3† for details). After this correction, the initial XA and XB signal amplitudes in NS–Pt are only 36% those of free NSs (Fig. 4b and S7†), indicating ultrafast electron decay that is faster than the instrument response time of our TA measurement (≪150 fs). This initial loss was followed by a XB recovery (much faster than free NSs) and a concomitant spectral evolution from the XB feature to a derivative-like feature within 300 ps (Fig. 4a upper panel). The derivative-like feature (Fig. 4a lower panel) can be attributed to the CS state (NS^+^–Pt^–^) generated by ET from the NS to the Pt nanoparticle, similar to the CS features observed in CdSe NS–MV^2+^ (Fig. 3a) and in CdS NR–Pt heterostructures.^20^

The formation and decay kinetics of the CS state were monitored at 577 nm (Fig. 4b). The signal at this wavelength in free NSs (Fig. 2b) showed a fast XA decay (0.41 ± 0.12 ps) and negligible TA signals afterwards. As shown in Fig. 4b and S7,† in NS–Pt, the XA feature shows a faster decay (0.18 ± 0.06 ps), consistent with an ultrafast exciton quenching process. After the ultrafast decay of the XA signal, the CS signal grew in at <300 ps due to charge separation and decayed at longer delay times due to charge recombination. Multiple exponential fit of the kinetics (Fig. 4b, see ESI† for details) revealed half-lives of 9.4 ± 0.7 ps and 75 ± 14 ns for charge separation and charge-recombination, respectively. Further support of the above assignment is provided by comparing the kinetics at 577 and 553 nm. Because the latter contains the contributions of XB bleach and CS signals, its decay kinetics should be identical to that at 577 nm after the completion of the charge separation process. As shown Fig. S5,† these signals decay with the same kinetics after 300 ps, reflecting the charge recombination processes. During the charge separation process, XB recovery should agree with the formation of CS, which is also shown in Fig. 4c. Here, we have scaled the kinetics at 577 nm and displaced it vertically to allow better comparison with the bleach recovery at 533 nm. Indeed, these two kinetics agree from ∼1.5 to ∼300 ps, indicating that exciton quenching after 1.5 ps is purely due to electron transfer. The amplitude of ∼553 nm at 1.5 ps is 13.4 ± 0.5% that of free NS (Fig. 4c). Since charge separation has negligible contribution within 1.5 ps, this ratio is approximately the charge separation yield in NS–Pt. The time-dependent populations of electron (XB) in NSs and CS states are shown in Fig. 4d.

The comparison above reveals that 87% of the excitons undergo ultrafast quenching (before 1.5 ps) without forming CS states. We attribute this pathway to rapid energy transfer from free excitons in the NS to Pt. As described in the ESI S8,† due to its large transition dipole, the energy transfer time of a free exciton in contact with the Pt particle (separated by ∼2.0 nm, the sum of the radii of the Pt nanoparticle and exciton in the CdSe NS) is estimated to be ∼29 fs. For excitons generated far away from the Pt, their quenching is limited by the exciton transport rate. Due to large in-plane exciton mobility, the average exciton quenching time is estimated to be ∼200 fs, which is in qualitative agreement with the observed half-life of the fast XB recovery component of <150 fs.

**Scheme 1:**
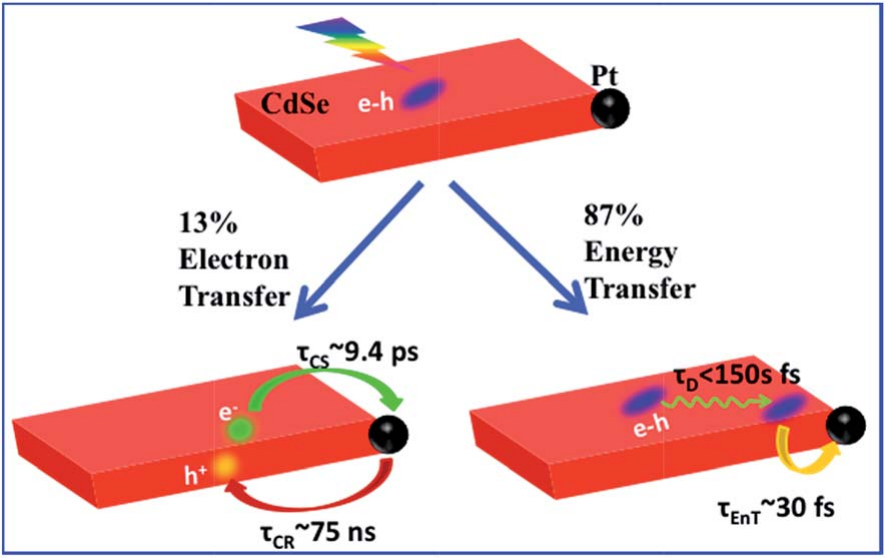
Competition between energy and electron transfer pathways in CdSe NS–Pt. Fast in-plane exciton mobility leads to fast energy transfer quenching. Trapped excitons can be dissociated by electron transfer to Pt.

The remaining 13.4 ± 0.5% of excitons was dissociated by ET to Pt, as shown in Scheme 1b. We attribute this to ultrafast hole trapping, which reduced the mobility of excitons and enabled effective competition of electron transfer with ultrafast energy transfer. The trapping of the hole also leads to a long-lived charge separated state (75 ± 14 ns). This assignment is supported by the presence of ultrafast hole trapping and long-lived conduction band electrons in free CdSe NSs. Similar hole-trapping induced long-lived charge separation has also been observed in CdS NR–Pt and CdS NR–Au.^20,47^ In the presence of hole acceptors, these long-lived charge separated states allow removal of trapped holes and accumulation of electrons in the Pt tip for H_2_ production.^16,17^ The key differences between CdSe NSs and CdS NRs are that in the latter, exciton quenching through diffusion and energy transfer is slow compared to hole trapping, leading to a near unity yield of charge separation in CdS NR–Pt.^20^ This difference can be attributed to atomically precise thickness in the quantum-confined dimension in NSs, which minimizes energy disorder and enables ultrafast exciton diffusion. This insight suggests that a possible way to improve the charge separation yield in NSs is to use nanosheet heterostructures with efficient hole localization at selective domains, such as the recently reported CdSe NS/CdS crown heterostructure where holes are confined in CdSe NS.^33,48^ Similar strategies have been successfully demonstrated in CdSe/CdS dot-in-rod nanorod heterostructures.^17,18^

## Conclusions

In conclusion, we have prepared Pt tipped CdSe nanosheets with well-defined morphology and strong interaction between the semiconductor and metal domains. The mechanisms of efficient exciton quenching in these heterostructures have been investigated by TA spectroscopy. The result revealed ultrafast quenching of 86.6% excitons (half-life <150 fs) by fast exciton transport to the NS–Pt interface followed by rapid energy transfer. The remaining 13.4% of excitons were first localized due to hole trapping and then dissociated through interfacial electron transfer (∼9.4 ps) from the CdSe NS to the Pt tip. The charge-separated state was long-lived (∼75 ns) as a result of the hole trapping, which provides potential for charge accumulation. To apply these NS–Pt heterostructures in solar driven H_2_ generation, the exciton dissociation yield needs to be improved, which can potentially be achieved by using nanosheet heterostructures with engineered hole traps.
