# Supplementary material for: Ultrafast exciton quenching by energy and electron transfer in colloidal CdSe nanosheet–Pt heterostructures
Source: Chem Sci. 2014 Nov 4;6(2):1049–54. doi: 10.1039/c4sc02994a (PMC5811111; doi:10.1039/c4sc02994a)
Supplement: Supplementary file 1 [file SC-006-C4SC02994A-s001.pdf]

Electronic Supplementary Material (ESI) for Chemical Science.
This journal is © The Royal Society of Chemistry 2014

Electronic Supplementary Information for:

Ultrafast Exciton Quenching by Energy and Electron Trnasfer in Colloidal CdSe Nanosheet-Pt Heterostructures

Kaifeng Wu, Qiuyang Li, Yongling Du, Zheyuan Chen, and Tianquan Lian

Department of Chemistry, Emory University, 1515 Dickey Drive, NE, Atlanta, GA 30322, USA.

Content list:

S1. Sample Synthesis

S2. Transient Absorption spectroscopy sep-ups

S3. Absorption spectrum fitting

S4. Single exciton experimental conditions

S5. Charge separation and recombination in CdSe NS-MV^{2+} complexes

S6. Kinetics fitting in free CdSe NS

S7. Kinetics fitting in CdSe NS-Pt

S8. Exciton quenching by diffusion and energy transfer in CdSe NS-Pt

S9. Estimation of hole trapping time scale in NS

S1. Sample Synthesis

Synthesis of CdSe nanosheets (NSs) CdSe NSs were synthesized following procedures reported in the literature, with slight modifications.[1-3] In a typical synthesis, 170mg of Cadmium myristate ($Cd(myr)_2$) and 15ml of 1-Octadecene (ODE) were mixed in a three-neck flask and degassed under vacuum for 1 hour at 100 ^oC. The mixture was then heated under argon flow at 240 ^oC and injected swiftly with 1ml of Selenium (Se) solution (0.15 M in ODE). After 10 seconds, 80mg of Cadmium acetate dihydrate ($Cd(Ac)_2 \cdot 2H_2O$) dispersed in ODE (in a slurry form) were introduced to initiate nanosheet formation. The reaction continued for 10 minutes at 240 ^oC before quenching by water bath. At room temperature,

1mL of oleic acid (OA) was added to the mixture for better dispersion of NSs. The product was a mixture of quantum dots (QDs) and NSs. NSs were separated from the QDs using centrifugation at 3000 rpm for 10 minutes. Final NSs were dispersed in chloroform for optical studies.

Synthesis of CdSe NS-Pt. Pt nanoparticle tipped CdSe NSs were synthesized in a similar way to a reported synthesis procedure for Pt tipped CdS nanorods.[4] Briefly, a mixture of 0.2 mL oleic acid (OA), 0.4 mL oleylamine (OLAm), 40 mg 1,2-hexadecanediol (HDT) and 10 mL diphenyl ether (DPE) was degassed under vacuum for 1 hour at 100 ^{o}C. After that, the temperature of the solution was raised to 195 ^{o}C under argon flow. 15 mg Pt acetylacetonate was added to a suspension of NSs in dichlorobenzene and sonicated for 5 minutes to dissolve the Pt precursor. This solution was swiftly injected into the DPE solution at 195 ^{o}C. The reaction was allowed to proceed for 3.5 min before quenched by water bath. The product was washed by precipitation with ethanol followed by centrifugation, and finally dispersed in chloroform. To independently obtain the absorption spectrum of Pt nanoparticles, free Pt nanoparticles were also synthesized under similar conditions without the addition of CdSe NSs. The growth time was longer, typically 15 minutes because the homogeneous nucleation of Pt nanoparticles was slower than the heterogeneous nucleation on the surfaces of CdSe NSs.

Transmission Electron Microscopy (TEM) images of CdSe NSs before and after Pt tip growth are shown in Figure S1a and 1b, respectively. The NSs had the tendency to stand up on the TEM grids and therefore exhibited rod-like shapes, but NS-Pt appeared to lay flat on the grid. ~2%, 95%, and 3% of NSs had 0, 1, and >1 Pt particles. Based on the observed complete PL quenching (Figure 1a), we believe that all NSs have Pt particles on them. The Pt particles showed a relatively broad size distribution, which suggested that there may exist small Pt clusters that were not counted.

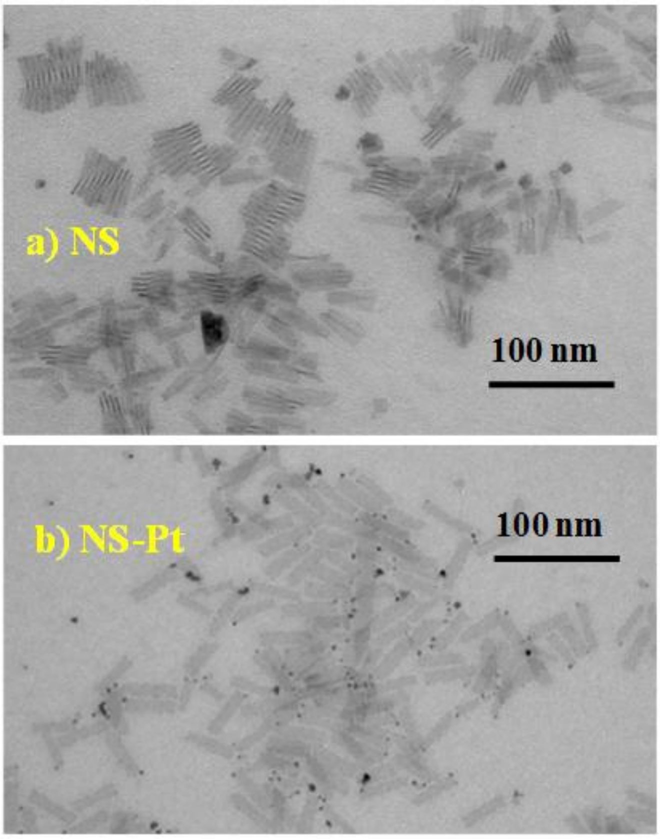

Figure S1. Transmission Electron Microscopy (TEM) images of CdSe NSs before (a) and after (b) the growth of Pt nanoparticles. The Pt particles appear to preferentially grow at the edge or vertex.

S2. Transient Absorption (TA) Spectroscopy Set-ups

Visible Femtosecond Transient Absorption. The femtoseconds transient absorption measurements were conducted in a Helios spectrometer (Ultrafast Systems LLC) with pump and probe beams derived from a regeneratively amplified Ti:sapphire laser system (Coherent Legend, 800 nm, 150 fs, 2.4 mJ/pulse, and 1 kHz repetition rate). One part of the 800 nm output pulse was used to produce the 400 nm pump beam by frequency-doubling in the BBO crystal. A series of neutral-density filter wheels were used to adjust the power the pump beam. The pump beam was focused at the sample with a beam waist of about 300 μm. A white light continuum (WLC) from 420 to 800 nm was generated by attenuating and focusing ~10 μJ of the 800 nm pulse into a sapphire window. The WLC was split into a probe and reference beam. The probe beam was focused with an Al parabolic reflector onto the sample (with a beam waist of 150 μm at the sample). The reference and probe beams were focused into a

fiber-coupled multichannel spectrometer with complementary metal-oxide-semiconductor (CMOS) sensors and detected at a frequency of 1 kHz. The intensities of the pump and probe beams were ratioed to correct for pulse-to-pulse fluctuation of the white-light continuum. The delay between the pump and probe pulses was controlled by a motorized delay stage. The pump beam was chopped by a synchronized chopper to 500 Hz. The change in absorbances for the pumped and unpumped samples was calculated. 1 mm cuvettes were used for all spectroscopy measurements. The instrument response function (IRF) of this system was measured to be ~160 fs by measuring solvent responses under the same experimental conditions (with the exception of a higher excitation power).

Nanosecond Transient Absorption. Nanosecond TA was performed with the EOS spectrometer (Ultrafast Systems LLC). The pump beam at 400 nm was generated in the same way as femtosecond TA experiments. The white light continuum (380-1700 nm, 0.5 ns pulse width, 20 kHz repetition rate) used here was generated by focusing a Nd:YAG laser into a photonic crystal fiber. The delay time between the pump and probe beam was controlled by a digital delay generator (CNT-90, Pendulum Instruments). The probe and reference beams were detected with the same multichannel spectrometers used in femtosecond TA experiments. The IRF of this system was measured to be ~280 ps. The data from femtosecond and nanosecond transient spectrometers overlap at delay time windows from 0.5 to 1 ns. To connect spectra and kinetics from these spectrometers, we scale the amplitudes of the nanosecond data by a factor to match the femtosecond results in the overlapping delay time windows.

Time-Resolved Fluorescence Lifetime Measurements. Time-correlated single photon counting (TCSPC) method was used to measure the fluorescence decay of the NSs. Samples were hold in a 1 cm cuvette and measured at the right angle geometry. The output pulses centered at 800 nm (~100 fs, 80 MHz) from a mode-locked Ti:Sapphire laser (Tsunami oscillator pumped by a 10 W Millennia Pro, Spectra-Physics) were passed through a pulse picker (Conoptics, USA) to reduce the repetition rate and then frequency-doubled in a BBO crystal to generate pump pulses at 400 nm and used to excite NSs. The emissions from NSs were detected by a microchannel-plate photomultiplier tube (Hamamatsu R3809U-51), the output of which was amplified and analyzed by a TCSPC board (Becker & Hickel SPC 600).

The IRF of this system was determined to be 240 ps by fitting the rising process of a long-lived fluorescent sample (CdSe@ CdS nanorods) at the same experiment condition.

S3. Absorption spectrum fitting

The ground state absorption spectrum of CdSe NSs can be fitted by a sum of multiple exciton bands, each represented by a Lorentzian function L_i (with peak height of H_i, center energy of E_i and half width of Γ_i), and a broad background function (*bg*). The use of Lorentzian instead of Gaussian is due to negligible inhomogeneous broadening in NS thicknesses. The origin of the background function is unclear but it could be Rayleigh scattering or unresolved transitions.[5] The ground state absorption spectrum of NS can be fit to the following function :

$$Abs_{NS}(\omega) = bg(\omega) + \sum_{i=1}^{4} \frac{H_i}{(\hbar\omega - E_i)^2 + \Gamma_i^2} \qquad \text{(S1).}$$

The fitting curve is shown in Figure S2a and the fitting parameters are listed in Table S1. The fitted background function is:

$$bg(\omega) = C \bullet (\hbar\omega - E_g)^{1/2}, \qquad \text{(S2)}$$

where Eg is the band gap of NS (2.2 eV) and C is a pre-factor.

The ground state absorption spectrum of CdSe NS-Pt can be approximately treated as superposition of NS absorption with Pt absorption Abs_{Pt} (ω). The latter was obtained from free Pt nanoparticles in chloroform. Therefore, we fit NS-Pt absorption spectrum according to the following equation:

$$Abs_{NS-Pt}(\omega) = Abs_{NS}(\omega) + Abs_{Pt}(\omega) = \left[bg(\omega) + \sum_{i=1}^{4} \frac{H_i}{(\hbar\omega - E_i)^2 + \Gamma_i^2} \right] + Abs_{Pt}(\omega) \qquad \text{(S3).}$$

The fitting curve is shown in Figure S2b and the fitting parameters are listed in Table S1. Comparison of the fitting parameters in CdSe NS and CdSe NS-Pt reveals that the exciton bands are red-shifted and broadened in the latter. The broadening is more prominent for lowest energy exciton band, which implies that the broadening results more likely from energy level dependent electronic interaction between the NS and Pt than dielectric environment change caused by the Pt..

The successful treatment of NS-Pt absorption as sum of NS and Pt absorptions allows us to determine the contribution of Pt absorption at excitation wavelength (400 nm) in our transient absorption (TA) experiments. Excluding Pt absorption, the NS absorption only accounts for 40.2 % of NS-Pt absorption at 400 nm. This factor will be used to scale the TA signal of free NS when compared with the TA signal of NS-Pt so as to correct for the number of photons absorbed by the NS. The justification for this scaling has been derived in our previous work.[6]

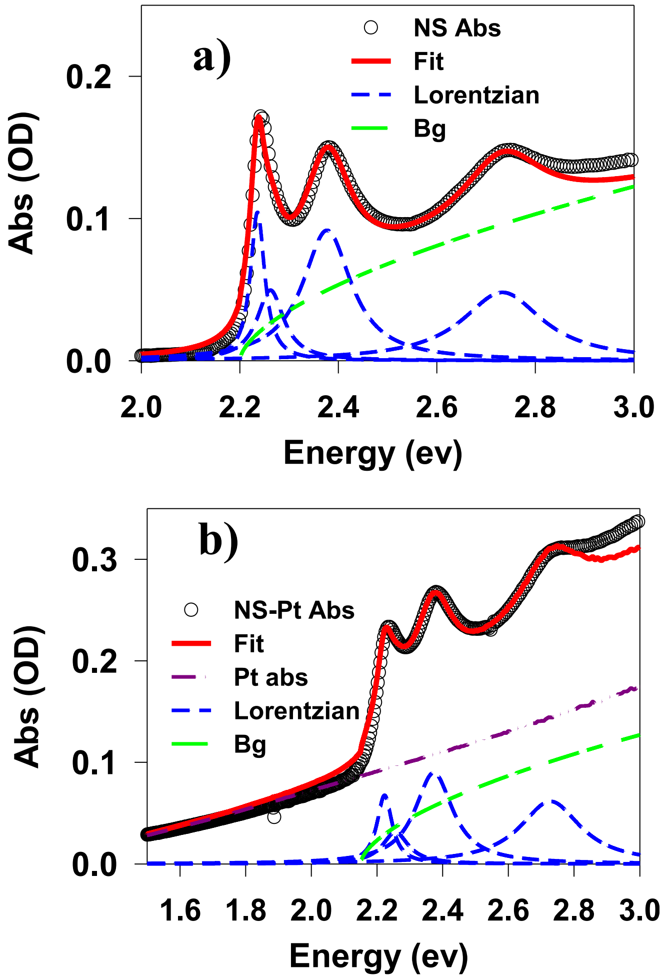

Figure S2. Fitting of the absorption spectra of a) CdSe NSs and b) CdSe NS-Pt, showing the absorption spectra (black open circles) and their fits (red solid line) by a sum of Lorentzian bands (blue dashed lines) and a background function (green dashed line) and Pt absorption (purple dashed line, in b only).

Table S1. Fitting parameters for Figure S2

	CdSe NS		CdSe NS-Pt	
	Center (eV)	Width (eV)	Center (eV)	Width (eV)
L1	2.236	0.035	2.223	0.056
L2	2.262	0.066	2.256	0.096
L3	2.377	0.120	2.373	0.140
L4	2.733	0.200	2.728	0.225

S4. Single exciton experimental conditions

Estimating average number of photo-generated excitons on each CdSe NS requires absorption cross section of these NSs, which is not measured in this work. However, to ensure that our experiments were performed under single exciton conditions, i.e. each NS has either one or zero exciton, we measured power-dependent exciton kinetics for these NSs. As shown in Figure S3, the kinetics at three indicated powers agree well with each other after scaling. Also, the initial signal amplitudes are proportional to excitation powers (inset). Therefore, these experiments were in the linear regime, or single exciton excitation conditions. All of our experiments shown in the main text correspond to excitation power of 19 uW.

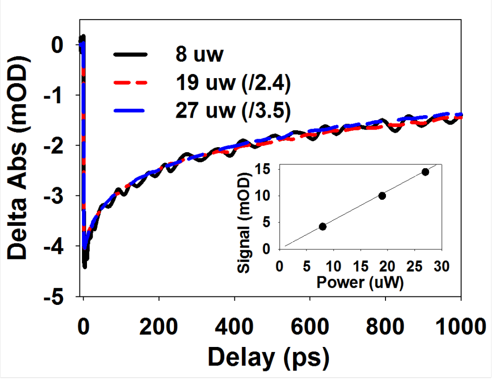

Figure S3. XB recovery kinetics at different excitation powers. They are scaled for better comparison and the scaling factors are labeled. The inset is a plot of initial signal amplitudes as a function of excitation power.

S5. Charge separation and recombination in CdSe NS-MV^{2+} complexes

The kinetics of $MV^{+\bullet}$ radical signal can be fitted with the following equation:

$$S_{radical}(t) = A_{radical}\left[\sum_{i=1}^{2} b_i e^{-t/\tau_{d,i}} - \sum_{i=1}^{3} a_i e^{-t/\tau_{f,i}}\right] \quad \text{(S4)},$$

where a_i (b_i) and $\tau_{f,i}$ ($\tau_{d,i}$) are the amplitude and time constant for i^{th} component of the radical formation (decay), respectively and A is the radical signal amplitude. Fitting parameters are shown in Table S2, form which charge separation and recombination half-lives are determined to be 7.7±1.1 ps and 3.1±1.2 ns, respectively. Accounting for XB lifetime in free NS (0.63 ns half life, Table S3), the charge separation yield in this complex is 98.8±0.02%.

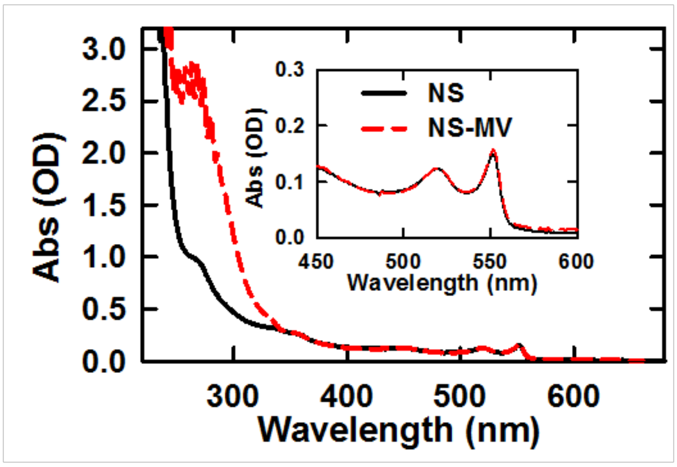

Figure S4. Absorption spectra of CdSe NSs and CdSe NS-MV^{2+} complexes.

Table S2. Fitting parameters for $MV^{+\bullet}$ radicals in CdSe NS-MV^{2+} complexes

	$\tau_{f,1}$/ps (a_1)	$\tau_{f,2}$/ps (a_2)	$\tau_{f,3}$ /ps (a_3)	$\tau_{1/2}$ /ps
CS	7.50±1.85 (77.6±2.6%)	21.6±3.4 (22.4±1.25%)	NA	7.7±1.1
	$\tau_{d,1}$/ns (b_1)	$\tau_{d,2}$/ns (b_2)	$\tau_{d,3}$ /ns (b_3)	$\tau_{1/2}$ /ns
CR	2.64±0.32 (76.9±3.3%)	64.0±9.3 (13.1±2.4%)	>>1000 (10.0±1.1%)	3.1±1.2

S6. Kinetics fitting in free CdSe NS

The kinetics of exciton bleach (XB) in Figure 2c was fitted to the following equation:

$$XB(t) = A_{XB}\left[\sum_{i=1}^{4} a_i e^{-t/\tau_i} - e^{-t/\tau_f}\right] \qquad \text{(S5)},$$

where τ_f is the formation rate of XB and a_i and τ_i are the amplitude and time constant for i^{th} component of XB decay, respectively. The fitting parameters are listed in Table S3.

Table S3. Fitting parameters for XB and PL of free NS

	$\tau_{f,1}$/fs	τ_1/ns (a_1)	τ_2/ns (a_2)	τ_3 /ns (a_3)	τ_4 /ns (a_4)	$\tau_{1/2}$ /ns
XB	80±27	0.108±0.009 (16.8±0.4%)	0.617±0.012 (50.9±0.5%)	10.9±0.3 (23.4±0.3%)	220±8 (8.9±0.2%)	0.63±0.03
	$\tau_{f,PL}$/ns	A_1	τ_{Htr}/ns	A_2	τ_{QD} /ns	$\tau_{1/2}$ /ns
PL	<<0.24 ns	91.9±0.7%	<<0.24 ns	8.1±0.4%	6.0±0.2	0.18±0.02

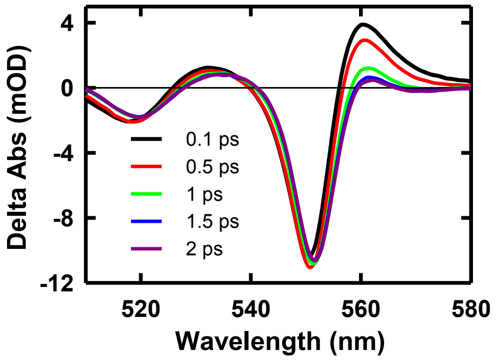

Figure S5. Spectral evolution (red-shifting) of 400 nm excited CdSe NSs within 2 ps.

The PL decay in Figure 2d is fitted with a constrained model. Specifically, we attribute the long-lived XB components (32.3%) to electrons recombining with trapped holes. This hole-trapping likely occurs on sub-ps time scale (see later discussions) and should lead to ultrafast PL decay. In addition, the 16.8% fast decay component of XB can be attributed to electron trapping, which should also lead to fast PL decay. The rest (50.9%) of XB decay occurs through radiative and nonradiative decay with holes. These percentages and time

scales are fixed in fitting PL decay. We also find that the PL kinetics has a long-lived component on ~10 ns time scale, likely resulting from some QDs species in the NSs. They can also be seen in the static emission spectrum (Figure 1b). Considering all of these components, the PL kinetics in Figure 2d is fitted to the following equation:

$$PL(t) = C\left[A_1[(a_3 + a_4)e^{-t/\tau_{Htr}} + a_1 e^{-t/\tau_1} + a_2 e^{-t/\tau_2}] + A_2 e^{-t/\tau_{QD}} - e^{-t/\tau_{f,PL}}\right] * IRF \quad \text{(S6)}.$$

Here, A1 and A2 are percentage of photons emitted by NSs and QDs, respectively. $\tau_{f,PL}$ and τ_{Htr} are formation time constant and hole trapping time constant, respectively. The other parameters are the same as in equation S5. Due to limited IRF (240 ps), convolution needs to be performed in the fitting process. As shown in Figure 2d, the PL kinetics can be well-fitted with this model, validating our understanding for carrier trapping kinetics in these NSs. The fitting parameters are listed in Table S3. According to the fitting, there are 8.1±0.4% photons emitted by QDs. They are not obvious on the TA spectrum because of their much smaller extinction coefficients compared with NSs.

S7. Kinetics fitting in CdSe NS-Pt

The kinetics at 577 nm (averaged over 573-580 nm) is fitted to the following equation:

$$S_{577nm}(t) = A_{CS,5770nm}\left[\sum_{i=1}^{3} b_i e^{-t/\tau_{CR,i}} - \sum_{i=1}^{3} a_i e^{-t/\tau_{cs,i}}\right] + A_{XA}\left[\sum_{i=1}^{3} c_i e^{-t/\tau_i} - e^{-t/\tau_f}\right] \quad \text{(S7)},$$

where a_i (b_i) and $\tau_{CS,i}$ ($\tau_{CR,i}$) are the amplitude and time constant for i^{th} component of the charge separation (recombination) process, respectively and $A_{CS,577nm}$ is the charge separated state signal amplitude at 573-580 nm. The second term on the right hand side of equation S7 is contributed by XA signal. Its amplitude and formation time constant are determined from the fit to the XA kinetics in free NSs. The fitting parameters are listed in Table S4, from which charge separation and recombination half lives are determined to be 9.4±0.7 ps and 75±14 ns, respectively.

Table S4. Fitting parameters charge separation/recombination in CdSe NS-Pt

	$\tau_{CS,1}$/ps (a_1)	$\tau_{CS,2}$/ps (a_2)	$\tau_{CS,3}$ /ps (a_3)	$\tau_{1/2}$ /ps
CS	8.26±1.07 (78.0±4.0%)	131±28 (22.0±4.7%)	NA	9.4±0.7

	$\tau_{CR,1}$/ns (b_1)	$\tau_{CR,2}$/ns (b_2)	$\tau_{CR,3}$ /ns (b_3)	$\tau_{1/2}$ /ns
CR	3.05±0.26 (44.5±1.5%)	208±16 (22.9±0.7%)	>>1000 (32.6±0.4%)	75±14
	τ_f/ps	τ_1/ps (c_1)	τ_2/ps (c_2)	$\tau_{1/2}$ /ps
XA	<<0.15	<<0.15 (59.3±2.5%)	0.30±0.05 (40.7±1.8%)	<0.15

The signal at 553 nm (averaged over 551-555 nm) is initially dominated by XB (A_{XB}) and latter (after ~300 ps) by charge separated state ($A_{CS,553\,\mathrm{nm}}$). The kinetics at this wavelength can be described by:

$$S_{553nm}(t) = A_{CS,553nm}[\sum_{i=1}^{3} b_i e^{-t/\tau_{CR,i}} - \sum_{i=1}^{3} a_i e^{-t/\tau_{cs,i}}] + A_{XB}\left[\alpha \sum_{i=1}^{3} a_i e^{-\frac{t}{\tau_{cs,i}}} + (1-\alpha) f_{EnT}(t)\right]$$

$$= A_{CS,553\,\mathrm{nm}} \sum_{i=1}^{3} b_i e^{-t/\tau_{CR,i}} + \left[(A_{XB} - A_{CS,553\,\mathrm{nm}}) \sum_{i=1}^{3} a_i e^{-\frac{t}{\tau_{cs,i}}}\right] \quad \text{(S8).}$$

We have assumed that exciton decay by two pathways: one part (α) proceeds by electron transfer to form the charge separated state and the remaining (1-α) undergoes energy transfer with a decay function $f_{Ent}(t)$.

Comparison of eq. S7 and S8 suggests that after the initial decay of XA feature at 577 nm and the fast energy transfer decay of XB at 553 nm, the kinetics of these wavelengths should be both due to charge separation and recombination. Indeed, when the charge separation process is completed (after ~ 300 ps), both the kinetics at 553 nm and 577nm contains only the charge recombination contribution and should agree with each other (with opposite sign), as shown in Figure S6. From 1.5 ps to 300 ps, the growth of kinetics at 577 nm agrees well with the XB recovery at 553nm, since both are due to charge separation, as shown in Figure 3b.

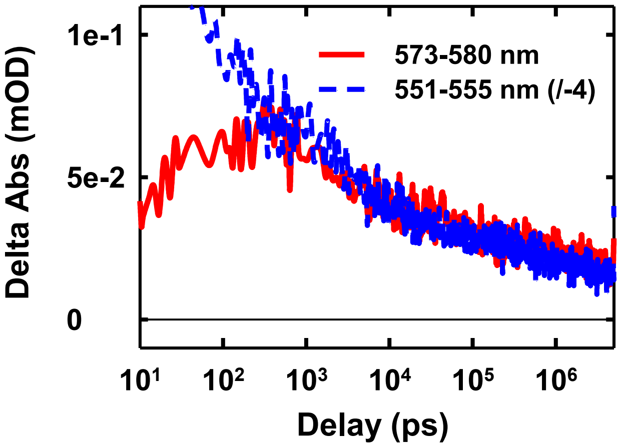

Figure S6. Comparison of kinetics at 551-555 nm (blue dashed line) and 573-580 nm (red solid line). The former has been reduced and inverted by a factor of -4 for better comparison of these signals of different signs.

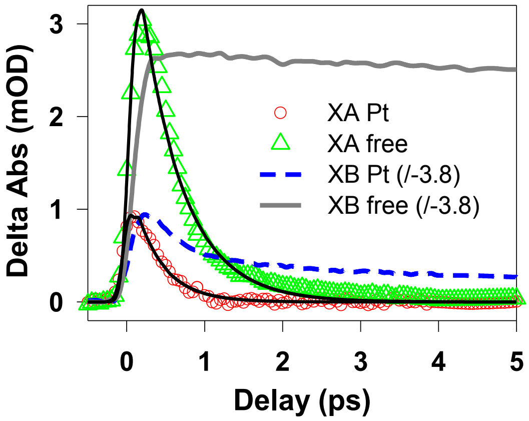

Figure S7. Comparison of early time kinetics at 577 nm in NS-Pt (red circles) and free NSs (green triangles). Also shown are the kinetics at 553 nm in NS-Pt (blue dashed line) and free NSs (gray solid line), which have been reduced and inverted by a factor of -3.8 for comparison.

S8. Exciton quenching by diffusion and energy transfer in CdSe NS-Pt

We assume that excitons in CdSe NS can be quenched by Pt through Forster Resonant Energy Transfer (FRET) mechanism. Previous studies have confirmed the applicability of

this model to these nanoscale objects even though the donor and/or acceptor dimensions are similar to their separations. [10,11]

The Forster radius, the distance between donor and acceptor at which the fluorescence quenching efficiency is 50%, is given by the following equation:[12,13]

$$R = 0.211[\kappa^2 \Phi_D J(\lambda)/n^4]^{1/6} \quad \text{(S12)},$$

in which κ^2 is dipole orientation factor. The general expression for κ^2 is given by:[12,13]

$$\kappa^2 = \langle(\cos\alpha - 3\cos\beta\cos\gamma)^2\rangle \quad \text{(S15)},$$

where α is the angle between the donor and acceptor transition moments, β is the angle between the donor moment and the line joining the centers of the donor and acceptor, and γ is the angle between the acceptor moment and the line joining the centers of the donor and acceptor. The transition dipoles in the spherical Pt nanoparticle can be assumed to be isotropic. The emission dipole in the NS is likely randomly-distributed in the plane, as demonstrated by the 2-D polarized emission in NSs. In this case, α, β, and γ are all allowed to range from 0 to . π and the κ^2 in equation S15 is averaged to 2/3 .

Φ_D is emission quantum yield of donor, $J(\lambda)$ is the overlap integral between donor emission and acceptor absorption spectra, and n is the refractive index of the medium. The overlap integral can be calculated using:

$$J(\lambda) = \frac{\int d\lambda I_D(\lambda)\varepsilon_A(\lambda)(\lambda)^4}{\int d\lambda I_D(\lambda)} \quad \text{(S13)},$$

where $I_D(\lambda)$ and $\varepsilon_A(\lambda)$ are donor emission spectrum and acceptor molar absorptivity, respectively. The molar absorptivity of Pt nanoparticles are estimated from the molar absorptivity of NS and the absorption spectrum of NS-Pt, by assuming 1:1 molar ratio of NS to Pt. The molar absorptivity of NS is estimated from NS-MV^{2+} electron transfer experiments where we assume that one electron in NS bleaches half of NS transition and after transfer the electron generates one $MV^{+\cdot}$ radical.[14,15]

With the Foster radius, the energy transfer time constant can be calculated as:

$$\tau_{EnT} = \tau_0(\frac{r}{R})^6 \quad \text{(S14)},$$

Where τ_0 is the donor excited state lifetime in the absence of the acceptor and r is the donor-acceptor distance. The overlap integral between CdSe NS emission and Pt absorption

is calculated to be 8.7×10^{17} $M^{-1}cm^{-1}nm^4$.

According to Table S3, we have 50.9% band edge electron-hole pairs undergo radiative and non-radiative recombination with a time constant of 617 ps. Since the QY of these NSs is 38%, the QY of these band edge electron-hole pairs is ~74.7%. The radiative life-time of band-edge exciton is then estimated to be ~826 ps. Therefore, the Forster radius calculated from equation S12 is 14.1 nm, using the refractive index of chloroform (1.49).

Since energy transfer rate is sensitive to donor-acceptor distance, we calculate two cases when the excitons are near the NS/Pt interface and at the center of NS, respectively, as shown in Figure S8. In the former case, accounting for the bulk Bohr radius for CdSe (4.9 nm[16]) and the diameter of Pt tip (3.1 nm), the approximate energy transfer distance is 6.45 nm. Using equation S14, the energy transfer time constant at this distance is calculated to be ~7.65 ps. This time changes drastically with used Bohr radius value. For example, if we assume a Bohr radius of 1 nm, the energy transfer time is 29 fs. Recognizing the tightly-bound nature of e-h pair in NS[3], a Bohr radius smaller than bulk value should be used. Based on these estimations, the energy transfer occurs on 10s of fs time scale at the NS/Pt interface. If the exciton is located at the center of NS, the energy transfer distance is 20.7 nm, corresponding to an energy transfer time constant of 8.4 ns.

The above estimate suggests that exciton quenching via energy transfer is likely limited by the transport excitons to the NS/Pt interface, as shown in Figure S8. The exciton transport time can be estimated by assuming that exciton in-plane motion can be described by diffusion. The drift mobility of electron μ_e and hole μ_h in bulk CdSe is reported to be ~720 $cm^2/V.s$[17] and ~75$cm^2/V.s$[17], respectively. Due to strong e-h binding in NS, coupled e-h pair (i.e. exciton) diffusion is expected. We calculate the center of mass drift mobility of exciton according to:[18]

$$\mu_X = \frac{m_e\mu_e + m_h\mu_h}{m_e + m_h} = 220\ cm^2/V.s \qquad (S15),$$

where m_e (0.13 m_0) and m_h (0.45 m_0)are effective mass of electron and hole in bulk CdSe, respectively. The exciton diffusion constant D_X is related to its mobility via:

$$D_X = \frac{\mu_X kT}{e} = 5.5\ cm^2/\ s \qquad (S16),$$

where k is Boltzmann constant, T is temperature and e is electron charge. Due to

randomly-distributed exciton positions on NS upon photon absorption, numerical simulations are required to reproduce the diffusion times of excitons in NS. Here we provide a qualitatively estimate of time scale of this process. We assume the exciton is at the center of NS with the distance between exciton and Pt being 20.7 nm. In the case of 2-D diffusion, $t_D \sim \frac{L^2}{4D} = 195$ fs. This estimated time scale qualitatively agrees with the value calculated from equation S11. Therefore, it is a reasonable model for exciton quenching in NS-Pt. From this model, the ultrafast exciton quenching can be attributed to both giant emission dipole induced fast energy transfer rate and fast in-plane exciton transport in NS.

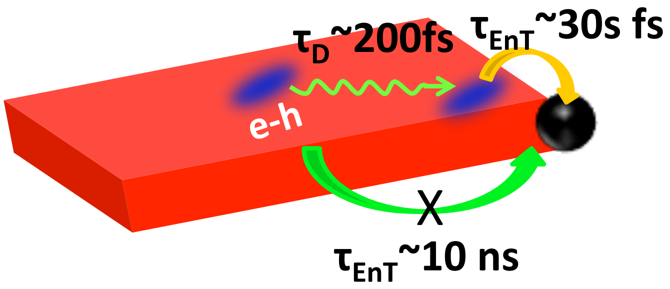

Figure S8. A scheme showing the mechanism of ***exciton diffusion controlled energy transfer***. Energy transfer from exciton near the nanosheet/Pt interface is fast. The overall exciton quenching rate is limited by in-plane diffusion, which is still on the ultrafast time scale due to large in-plane exciton mobility.

S9. Estimation of hole trapping time scale in NS

Previously, we have derived that 32.3±0.5% of holes are trapped on the surfaces on a << 0.24 ps time scale. However, the accurate measurement of hole trapping time is limited by time-resolution of PL decay and lack of obvious hole signals on TA spectra. According to our exciton quenching model in NSs, only NSs with hole trapping channels can transfer electron to Pt and the exciton dissociation yield is determined by the competition between hole trapping and energy transfer. Since we have estimated the time scale of energy transfer τ_{EnT} to be ~200 fs we can approximately calculate the time scale of hole trapping τ_{Htr} according to the following equation:

$$13.4 \pm 0.5\% = 32.3 \pm 0.5\% \left[\frac{\tau_{EnT}}{\tau_{EnT}+\tau_{Htr}}\right] \qquad \text{(S11).}$$

τ_{Htr} is estimated be ~0.28 ps. This time scale is similar to the ultrafast XA decay (0.41±0.12

ps) in free NSs. Therefore, it is likely that the XA decay in CdSe NSs is caused by the hole-trapping process.

References for SI:

(1) Ithurria, S.; Dubertret, B.: Quasi 2D Colloidal CdSe Platelets with Thicknesses Controlled at the Atomic Level. *J. Am. Chem. Soc.* **2008**, *130*, 16504-16505.

(2) Ithurria, S.; Bousquet, G.; Dubertret, B.: Continuous Transition from 3D to 1D Confinement Observed during the Formation of CdSe Nanoplatelets. *J. Am. Chem. Soc.* **2011**, *133*, 3070-3077.

(3) Ithurria, S.; Tessier, M. D.; Mahler, B.; Lobo, R. P. S. M.; Dubertret, B.; Efros, A. L.: Colloidal nanoplatelets with two-dimensional electronic structure. *Nat Mater* **2011**, *10*, 936-941.

(4) Habas, S. E.; Yang, P.; Mokari, T.: Selective Growth of Metal and Binary Metal Tips on CdS Nanorods. *J. Am. Chem. Soc.* **2008**, *130*, 3294-3295.

(5) Trinh, M. T.; Sfeir, M. Y.; Choi, J. J.; Owen, J. S.; Zhu, X.: A Hot Electron–Hole Pair Breaks the Symmetry of a Semiconductor Quantum Dot. *Nano Lett.* **2013**, *13*, 6091-6097.

(6) Wu, K.; Rodríguez-Córdoba, W. E.; Yang, Y.; Lian, T.: Plasmon-Induced Hot Electron Transfer from the Au Tip to CdS Rod in CdS-Au Nanoheterostructures. *Nano Lett.* **2013**, *13*, 5255-5263.

(7) Klimov, V. I.: Optical Nonlinearities and Ultrafast Carrier Dynamics in Semiconductor Nanocrystals. *J. Phys. Chem. B* **2000**, *104*, 6112-6123.

(8) Zhu, H.; Lian, T.: Enhanced Multiple Exciton Dissociation from CdSe Quantum Rods: The Effect of Nanocrystal Shape. *J. Am. Chem. Soc.* **2012**, *134*, 11289-11297.

(9) Jiang, Z.-J.; Kelley, D. F.: Hot and Relaxed Electron Transfer from the CdSe Core and Core/Shell Nanorods. *J. Phys. Chem. C* **2011**, *115*, 4594-4602.

(10) Curutchet, C.; Franceschetti, A.; Zunger, A.; Scholes, G. D.: Examining Forster energy transfer for semiconductor nanocrystalline quantum dot donors and acceptors. *J. Phys. Chem. C* **2008**, *112*, 13336-13341.

(11) Li, M.; Cushing, S. K.; Wang, Q.; Shi, X.; Hornak, L. A.; Hong, Z.; Wu, N.: Size-Dependent Energy Transfer between CdSe/ZnS Quantum Dots and Gold Nanoparticles. *J. Phys. Chem. Lett.* **2011**, *2*, 2125-2129.

(12) Stryer, L.: Fluorescence Energy Transfer as a Spectroscopic Ruler. *Annu. Rev. Biochem.* **1978**, *47*, 819-846.

(13) Clegg, R. M.: Fluorescence resonance energy transfer. *Curr. Opin. Chem. Biol.* **1995**, *6*, 103-110.

(14) Wu, K.; Liu, Z.; Zhu, H.; Lian, T.: Exciton Annihilation and Dissociation Dynamics in Group II–V Cd3P2 Quantum Dots. *J. Phys. Chem. A* **2013**, *117*, 6362-6372.

(15) Yang, Y.; Rodríguez-Córdoba, W. E.; Lian, T.: Ultrafast charge separation and recombination dynamics in lead sulfide quantum dot-methylene blue complexes probed by electron and hole intraband transitions. *J. Am. Chem. Soc.* **2011**, *133*, 9246-9249.

(16) Meulenberg, R. W.; Lee, J. R. I.; Wolcott, A.; Zhang, J. Z.; Terminello, L. J.; van Buuren, T.: Determination of the Exciton Binding Energy in CdSe Quantum Dots. *ACS Nano* **2009**, *3*, 325-330.

(17) Canali, C.; Nava, F.; Ottaviani, G.; Paorici, C.: Hole and electron drift velocity in CdSe at room temperature. *Solid State Communications* **1972**, *11*, 105-107.

(18) Luer, L.; Hoseinkhani, S.; Polli, D.; Crochet, J.; Hertel, T.; Lanzani, G.: Size and mobility of excitons in (6, 5) carbon nanotubes. *Nat Phys* **2009**, *5*, 54-58.
